# ONLINE-TICS: Internet-Delivered Behavioral Treatment for Patients with Chronic Tic Disorders

**DOI:** 10.3390/jcm11010250

**Published:** 2022-01-04

**Authors:** Martina Haas, Ewgeni Jakubovski, Katja Kunert, Carolin Fremer, Nadine Buddensiek, Sebastian Häckl, Martina Lenz-Ziegenbein, Richard Musil, Veit Roessner, Alexander Münchau, Irene Neuner, Armin Koch, Kirsten Müller-Vahl

**Affiliations:** 1 Department of Psychiatry, Social Psychiatry and Psychotherapy, Hannover Medical School, 30625 Hannover, Germany; haas.martina@mh-hannover.de (M.H.); jakubovski.ewgeni@mh-hannover.de (E.J.); katja.kunert@gmx.net (K.K.); fremer.carolin@mh-hannover.de (C.F.); nadine.buddensiek@gmx.de (N.B.); lenz-ziegenbein.martina@mh-hannover.de (M.L.-Z.); 2 Institute for Biostatistics, Hannover Medical School Hannover, 30625 Hannover, Germany; haeckl.sebastian@mh-hannover.de (S.H.); koch.armin@mh-hannover.de (A.K.); 3 Department of Psychiatry and Psychotherapy, Ludwig-Maximilians-University Munich, 80539 Munich, Germany; richard.musil@med.uni-muenchen.de; 4 Department of Child and Adolescent Psychiatry and Psychotherapy, Technische Universität Dresden, 01307 Dresden, Germany; veit.roessner@uniklinikum-dresden.de; 5 Institute of Systems Motor Science, University of Lübeck, 23562 Lübeck, Germany; alexander.muenchau@neuro.uni-luebeck.de; 6 Department of Psychiatry, Psychotherapy, and Psychosomatics, School of Medicine, RWTH Aachen University, 52074 Aachen, Germany; ineuner@ukaachen.de; 7 Institute of Neuroscience and Medicine 4 (INM-4), Forschungszentrum Jülich, 52428 Jülich, Germany; 8 JARA-BRAIN—Translational Medicine, 52056 Aachen, Germany

**Keywords:** Tourette syndrome, tics, Comprehensive Behavioral Intervention for Tics (CBIT), Internet-Delivered Comprehensive Behavioral Intervention for Tics (iCBIT), habit reversal training (HRT), tele-health

## Abstract

Comprehensive Behavioral Intervention for Tics (CBIT) is considered a first-line therapy for tics. However, availability of CBIT is extremely limited due to a lack of qualified therapists. This study is a multicenter (*n* = 5), randomized, controlled, observer-blind trial including 161 adult patients with chronic tic disorders (CTD) to provide data on efficacy and safety of an internet-delivered, completely therapist-independent CBIT intervention (iCBIT Minddistrict^®^) in the treatment of tics compared to placebo and face-to-face (f2f) CBIT. Using a linear mixed model with the change to baseline of Yale Global Tic Severity Scale-Total Tic Score (YGTSS-TTS) as a dependent variable, we found a clear trend towards significance for superiority of iCBIT (*n* = 67) over placebo (*n* = 70) (−1.28 (−2.58; 0.01); *p* = 0.053). In addition, the difference in tic reduction between iCBIT and placebo increased, resulting in a significant difference 3 (−2.25 (−3.75; −0.75), *p* = 0.003) and 6 months (−2.71 (−4.27; −1.16), *p* < 0.001) after the end of treatment. Key secondary analysis indicated non-inferiority of iCBIT in comparison to f2f CBIT (*n* = 24). No safety signals were detected. Although the primary endpoint was narrowly missed, it is strongly suggested that iCBIT is superior compared to placebo. Remarkably, treatment effects of iCBIT even increased over time.

## 1. Introduction

Chronic tic disorders (CTD) are neuropsychiatric disorders with childhood onset characterized by sudden, rapid, recurrent, non-rhythmic movements or vocalizations persisting for more than one year [1]. In addition to chronic motor and chronic vocal tic disorders (characterized by the occurrence of only motor or vocal tics), Tourette syndrome (TS) is defined as a chronic combined multiple motor and vocal tic disorder. The prevalence of TS in childhood is estimated to be about 0.5% and, in most patients, symptoms persist into adulthood, although often with a very low severity [2,3]. However, a small but relevant proportion of adult patients still suffers from impairing tics [2], resulting in reduced quality of life [4]. In addition, TS is a cost-intensive disease that causes high direct and in particular high indirect costs [5].

Cognitive behavioral therapy with habit reversal training (HRT) or Comprehensive Behavioral Intervention for Tics (CBIT) has been shown to be an effective and safe treatment for tics in children and adults [6,7]. CBIT is a 10-week manualized behavioral therapy consisting of eight sessions (plus optional booster sessions) including psychoeducation, HRT, functional analysis, and relaxation training [8]. Currently, HRT/CBIT is considered a first-line intervention for tics in patients with CTD/TS [9,10,11]. Despite this clear recommendation, in most countries, HRT/CBIT cannot be provided to patients because of a considerable lack of psychotherapists trained in and offering HRT/CBIT [10,12]. Thus, an unacceptably large number of patients never get the chance to be treated with HRT/CBIT, or at best have to accept very long waiting periods.

To overcome this shortage, we developed a completely therapist-independent internet-delivered platform to deliver CBIT (iCBIT Minddistrict^®^, Minddistrict GmbH, 10117 Berlin, Germany) for adult patients with CTD/TS. The platform was created by a team of experts (K.M.V., N.B., E.J.) and reviewed by one of the authors of the original face-to-face (f2f) CBIT manual (Sabine Wilhelm) [8]. Besides mode of delivery (internet vs. f2f), iCBIT follows exactly the manual for f2f CBIT developed by Woods et al. [8] with respect to number and content of treatment sessions, distribution of CBIT elements to the sessions, and duration of treatment. To enable successful use of iCBIT without support from a therapist, several descriptions, examples, and assistance were implemented, including videos from patients and experts, video animations, and a detailed FAQ section. For example, to improve the awareness training, detailed information on premonitory urges were provided, including examples, descriptions from other patients, and support that was experienced as helpful by others. For tic assessment, patients were instructed not only to list their tics, but to record themselves on video, watch themselves in a mirror, and to ask relatives to list all tics observed to improve tic awareness. With respect to the competing response training, concrete suggestions for possible competing responses were made including incompatible, but also alternative behaviors. Identical to the CBIT manual [8], in addition, a detailed description was given as to how to implement and practice the training and how to proceed in case of difficulties. For relaxation training, text descriptions and audios were provided. Finally, assistance for any technical problems was made available. (For further details about iCBIT, please refer to Jakubovski et al. [13]).

The present study was designed to examine efficacy and safety of this newly developed iCBIT in a large multicenter, prospective, controlled, randomized, observer-blind clinical trial comparing iCBIT to both placebo and f2f CBIT. Patients in the placebo group received an internet-delivered psychoeducation including detailed information on CTD/TS including historical aspects, phenomenology of tics and comorbidities, genetics, pathology, and treatment (excluding information on behavioral therapy for tics) using a similar platform as for iCBIT (including text, videos, and FAQ). F2f CBIT followed the CBIT manual by Woods et al. [8].

We hypothesized that iCBIT is superior to placebo and not inferior to f2f CBIT in the reduction of tics. In addition, we examined the course of tic severity during follow-up up to 6 months after end of treatment, influence on treatment on comorbid symptoms, and quality of life, as well as quality of the therapeutic alliance, depending on the mode of delivery of CBIT.

## 2. Materials and Methods

For detailed information on materials and methods additional to the information presented below, please refer to Jakubovski et al. [13].

### 2.1. Study Design

The present study was a prospective, multicenter, randomized, three-arm, controlled, observer-blind trial involving 5 study centers across Germany (Hannover Medical School (MHH), University of Munich, University of Dresden, University of Lübeck, University of Aachen). Due to the lack of appropriately trained therapists, f2f CBIT could reliably be provided only at MHH for the whole study period, and therefore a f2f CBIT arm was planned and realized only in this single center. Accordingly, in the center at MHH, participants were randomized with a 1:1:1 ratio to an 8 week treatment of either iCBIT, placebo, or f2f CBIT, whereas in all other 4 centers, participants were randomized with a 1:1 ratio to an 8 week treatment of iCBIT or placebo.

This study was observer-blind. Although—except for f2f CBIT—patients were not directly informed about treatment allocation after randomization, it can be assumed that participants were able to deduce to which group (iCBIT vs. placebo) they had been assigned to on the basis of the information provided. To avoid unblinding of the raters, various precautions were taken (e.g., strict separation between clinical raters and therapists, who performed f2f CBIT (only at MHH), and raters, who performed video tic assessments). For a more detailed description of precautions undertaken to prevent unblinding, please refer to Jakubovski et al. [13].

All participants randomized into the placebo or f2f CBIT groups were given the chance to gain access to iCBIT after completion of individual last follow-up visit.

In addition to 8 regular treatment sessions, patients in the f2f CBIT group were able to receive 2 optional booster sessions. During follow-up, patients in the iCBIT and placebo (after completion of individual last follow-up visit) groups had unlimited access to the platform, including content of all sessions.

There were 5 clinical visits: screening and baseline (if possible combined to one visit) (V1), 5 weeks after start of treatment (V2), 1 week after end of treatment (V3), and 2 follow-up visits at 3 (V4) and 6 months (V5) after the end of treatment. In addition, 2 telephone visits were carried out at weeks 17 and 29 (1.5 and 4.5 months, respectively, after end of treatment) to improve adherence, but without performing any clinical assessments.

The technical implementation of iCBIT as well as internet-delivered placebo was being set in place in cooperation with the Minddistrict GmbH, Friedrichstraße 100, 10117 Berlin, Germany (https://www.minddistrict.com (accessed on 30 November 2021)).

The study protocol was approved by all institutional review boards and written informed consent was obtained from all patients.

This study was registered on ClinicalTrials.gov Identifier: NCT02605902.

### 2.2. Eligibility Criteria

Participants needed to be adults (age ≥ 18 years) and must have had a main diagnosis of CTD or TS according to the DSM-5. At baseline, the minimum total tic score of the Yale Global Tic Severity Scale (YGTSS–TTS) must be 14 for patients with TS and 10 for patients with other CTD. In addition, a minimum score on the Clinical Global Impression scale for severity (CGI-S) of 4 was required. Further, anti-tic medication must remain on a stable dose for at least 6 weeks before entering the study. Patients needed to be sufficiently fluent in the German language and should not have had a history of schizophrenia or pervasive developmental disorders, or a comorbid condition in primary need of therapy. Patients with previous trials of behavioral treatment for tics including CBIT or HRT elements were excluded. For more detailed information on eligibility criteria, please refer to Jakubovski et al. [13].

### 2.3. Outcome Measures

The primary outcome measure was the YGTSS–TTS, with the primary endpoint being 1 week after end of treatment. Secondary outcome measures included the YGTSS–TTS at follow up, the Modified Rush Video-Based Tic Rating Scale (MRVS) [14], the Adult Tic Questionnaire (ATQ) [15], the Gilles de la Tourette Syndrome-Quality of Life Scale (GTS-QoL) (including the Quality of Life-Visual Analogue Scale (GTS-QoL-VAS) [16], and the Premonitory Urge of Tics Scale (PUTS-9) [17] at all time points. In addition, the CGI-S and Improvement Scores (CGI-I) [18] were assessed. Comorbid conditions and symptoms were assessed using specific scales (Yale-Brown Obsessive Compulsive Scale (Y-BOCS) [19,20], Conners’ Adult ADHD Rating Scales (CAARS) [21], Beck Depression Inventory-II (BDI-II) [22], and Beck Anxiety Inventory (BAI) [23]). The Working Alliance Inventory-Short Revised (WAI-SR) [24] was used to assess the therapeutic alliance with either the therapist or the internet platform. For more details, please refer to Jakubovski et al. [13].

### 2.4. Data Analysis

Analyses were performed in the intention-to-treat (ITT) population, taking into account all patients who were randomized to 1 of the 3 groups at baseline. Since a mixed model was primarily used, missing values did not have to be replaced considering the model, assuming that missing values were missing randomly. In the context of secondary and sensitivity analyses, missing values in the ITT population were replaced using last-observation-carried-forward (LOCF).

Sensitivity analyses were conducted in the per-protocol (PP) population, which included only those participants considered as compliant. Compliance was supposed, if patients participated properly in all clinical visits and treatment sessions defined as participation in at least 2 sessions, and—if less than 8 sessions were used—if in the opinion of the therapist the participant had reached the best possible treatment result. For a more detailed description of the data analysis, please refer to Jakubovski et al. [13].

Baseline characteristics with respect to sociodemographic variables and clinical assessments were compared between groups using the chi^2^ test for categorical variables and *t*-test for continuous variables. All analyses were carried out using SAS 9.4.

#### 2.4.1. Primary Analysis

In the primary analysis, superiority of iCBIT over placebo was analyzed in a linear mixed model for repeated measures with the change to baseline of tic severity (as assessed by YGTSS-TTS) as a dependent variable. Treatment, YGTSS-TTS at baseline, visit, center, anti-tic medication, and treatment-by-visit interaction were included as fixed effects. Patient was included as random effect, assuming a compound symmetric covariance pattern to model the within-patient errors. Restricted maximum likelihood (REML)-based approach in combination with the iterative Newton–Raphson algorithm was used to obtain estimates. Between–within method was applied for the estimation of the denominator degrees of freedom. The primary outcome variable was the difference in mean YGTSS-TTS change at V3 (1 week after end of treatment) compared to baseline between treatments (iCBIT minus placebo).

Significance tests were based on least square (LS) means using a two-sided α = 0.05 (two-sided 95% confidence interval (CI)). For a more detailed description, please refer to Jakubovski et al. [13].

#### 2.4.2. Secondary Analyses

The key secondary analysis was embedded into a hierarchical testing strategy and included non-inferiority testing of iCBIT to f2f CBIT using a mixed model equivalent to the primary analysis.

Further secondary analyses including the comparison of f2f CBIT and placebo at V3, as well as comparisons of the course of the YGTSS-TTS and other subscores of the YGTSS (YGTSS-motor tic score (MTS), YGTSS-vocal tic score (VTS), and YGTSS-impairment score) after end of treatment between all study groups were conducted with a model equivalent to the primary analysis.

In addition, responder analysis was carried out using a logistic regression model with the YGTSS-TTS as the dependent variable. Originally, response was defined by a 30% tic decrease at V3 (1 week after the end of treatment) [13]. However, following the recommendation by Jeon et al. [25], at a blind review meeting, the response criterion was modified and set at a 25% decrease at V3. Treatment, YGTSS-TTS at baseline, previous anti-tic medication, and center were included as independent variables. Wald test was used for significance testing, and odds ratios (95% CI) were computed for treatment comparison (iCBIT vs. placebo). The same analyses were also performed at V4 (3 months after end of treatment) and V5 (6 months after end of treatment).

Moreover, responder analysis was planned with the CGI-I as dependent variable and a response criterion defined by an improvement of 1–2 = “much” or “very much” improved.

Furthermore, secondary analyses included the analyses of further outcome variables assessing self- (ATQ) and video-rated (MRVS) tic severity, premonitory urges (PUTS-9), comorbid symptoms (Y-BOCS, CAARS, BAI, BDI-II), and disease severity (CGI-S), as well as quality of life (GTS-QoL, GTS-QoL-VAS) and therapeutic alliance (WAI-SR). The analyses were carried out according to the primary analysis, with the secondary outcome variable as the dependent variable. WAI-SR scores between treatment groups were compared for each visit using a linear regression model adjusted for study site and concomitant anti-tic medication.

#### 2.4.3. Safety Analyses

Number and kind of incidents were documented via open questions and were analyzed descriptively for the entire study population as well as separately for each study group (absolute and relative frequencies). In addition, frequencies of incidents were compared between the study groups using chi^2^ tests.

## 3. Results

We included 161 participants (112 males, 49 females, mean age = 35.66 years (SD = 12.47), range = 18–62 years) with CTD/TS according to DSM-5 between 29 September 2016 and 14 February 2020 across all study sites. Of these, 67 participants were allocated to iCBIT, 70 to placebo, and 24 to f2f CBIT (only at MHH). As shown in Table 1, there were no significant group differences on sociodemographic variables. Altogether, 40.4% of patients received anti-tic medication, with no significant differences between groups (iCBIT: 40.3%, placebo: 38.6%, f2f CBIT: 45.8%). At baseline, there were no differences between the iCBIT and placebo group regarding clinical characteristics for tics and comorbidities (for further details, please refer to Table 2).

Of 161 participants, 108 (67.1%) were considered as compliant until V3. Rate of non-compliance was lowest in the placebo group (22.9%) and similarly high in both treatment groups (iCBIT: 40.3%, f2f CBIT: 41.7%). Non-compliance could be inferred from missing clinic visits until V3 (*n* = 42) and non-participation in a sufficient number of iCBIT, placebo, or f2f CBIT treatment sessions (*n* = 11). In addition, during follow-up (clinic visits V4 and V5), a further nine patients dropped out.

### 3.1. Efficacy of iCBIT Compared to Placebo

Considering the adjusted LS means from the primary mixed model, the iCBIT group showed a larger tic reduction (as assessed by YGTSS-TTS) (2.54 (−3.53; −1.55)) in comparison to the placebo group (−1.26 (−2.16; −0.35)) at V3.

In the primary analysis, the difference in YGTSS-TTS change to baseline between placebo and iCBIT was −1.28 (−2.58; 0.01). Thus, the significance for superiority of iCBIT was narrowly missed and the null hypothesis could not be rejected as the upper 95% CI limit was marginally above 0. However, we found a very strong trend towards significance (*p* = 0.053). Results of sensitivity (including analysis in the PP population as well as ANCOVAs) analyses were in line with the primary analysis.

**Figure 1:**
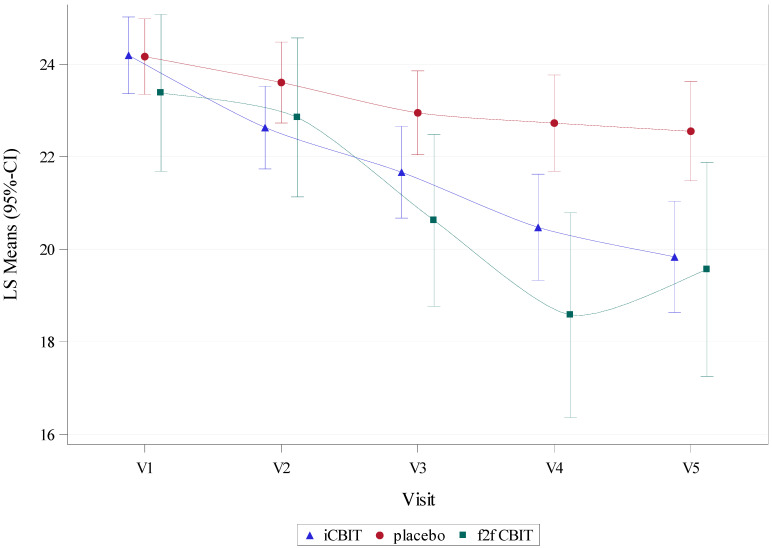
YGTSS-TTS at baseline (V1), during (V2 and V3), and after end of treatment (V4 and V5) in iCBIT compared to f2f CBT and placebo groups. LS means (iCBIT and placebo) for V1–V3 were derived from a mixed model for repeated measures (MMRM) including data for V1–V3 only and excluding patients of the f2f CBIT group (primary analysis model). LS means (iCBIT and placebo) for V4 and V5 each were derived from separate MMRMs including additional data up to V4 and V5, respectively. LS means (f2f CBIT) for V1–3 were derived from a MMRM, including data for V1–V3 only and excluding placebo patients (key secondary analysis model). LS means (f2f CBIT) for V4 and V5 each were derived from separate MMRMs, including additional data up to V4 and V5, respectively.

During follow-up, the difference in YGTSS-TTS reduction between iCBIT and placebo further increased and reached statistical significance at both V4 (−2.25 (−3.75; −0.75) *p* = 0.003) and V5 (−2.71 (−4.27; −1.16), *p* < 0.001) (for further details, please refer to Table 3 and Figure 1).

When comparing treatment effects on motor and vocal tics separately, we observed differences between iCBIT and placebo for motor tics at V3 (YGTSS-MTS: −0.89 (−1.59; −0.19) *p* = 0.013), V4 (−1.88 (−2.68; −1.08), *p* < 0.001), and V5 (−1.71 (−2.56; −0.86), *p* = 0.001), but none for vocal tics (YGTSS-VTS). Furthermore, the YGTSS-impairment scale indicated a significant improvement in the iCBIT group compared to placebo at V3 (−3.43 (−6.29; −0.56), *p* = 0.019) and V5 (−5.13 (−8.72; 1.54), *p* = 0.005) (for further details, please refer to Table 3 and Figures S1–S3).

The responder analysis for an at least 25% YGTSS-TTS improvement showed a difference in response rate of 15.2% between iCBIT and placebo at V3, which was statistically significant (*p* = 0.011). The difference slightly decreased 3 months (V4) after the end of treatment (12.5%; *p* = 0.062) and increased again at 6 months (V5) after the end of treatment (15.3%, *p* = 0.012). 

Responder analyses with the CGI-I as response variable could not be performed (i.e., the model does not converge as infinite maximum likelihoods are computed) due to the small number of participants fulfilling the response criteria of an improvement of 1–2 = “much” or “very much” improved.

### 3.2. Efficacy of iCBIT Compared to f2f CBIT

Since our primary endpoint narrowly missed statistical significance, the key secondary analysis was tested in an exploratory instead of a confirmatory way. Difference in YGTSS-TTS change to baseline between iCBIT and f2f CBIT at V3 was 0.98 [−1.01; 2.96]. Thus, since the upper bound of the 95% CI was below the non-inferiority margin of 3; non-inferiority of iCBIT in comparison to f2f CBIT could be observed in the ITT population.

In addition, during follow-up, no significant differences in YGTSS-TTS change to baseline were detected between iCBIT and f2f CBIT (for further details, please refer to Table 3 and Figure 1).

There were no significant differences between iCBIT and f2f CBIT when comparing change to baseline of YGTSS-MTS, -VTS, and -impairment separately, neither at V4 nor at V5 (for further details, please refer to Table 3).

### 3.3. Efficacy of f2f CBIT Compared to Placebo

Although the greatest tic reduction according to YGTSS-TTS was found after f2f CBIT, the difference between f2f CBIT and placebo did not reach statistical significance (1.26 (−0.59; 3.1) *p* = 0.180) at V3. The difference in YGTSS-TTS reduction between f2f CBIT and placebo further increased from V3 to V4 and reached significance (2.48 (0.36; 4.6); *p* = 0.022) but decreased again from V4 to V5 (1.33 (−0.85; 3.52); *p* = 0.230) (for further details, please refer to Table 3).

A similar course was seen for YGTSS-MTS, but not for YGTSS-VTS and YGTSS-impairment (for further details, please refer to Table 3).

### 3.4. Effects on Tics (According to Self and Video Assessments), Premonitory Urges, Comorbidities, Quality of Life, and Therapeutic Alliance

ICBIT was associated with a higher reduction of the ATQ sum score compared to placebo at V3 (−5.01 (−9.61; −0.41), *p* = 0.033) and V4 (−6.22 (−11.06; −1.39), *p* = 0.012), while at V5, there was a clear trend towards significance (−5.1 (−10.27; 0.08), *p* = 0.053). In addition, we observed a significant difference between iCBIT and placebo in tic reduction as assessed by MRVS at V4 (−1.40 (−2.40; −0.40), *p* = 0.006), a trend towards significance at V3 (−0.88 (−1.84; 0.08), *p* = 0.072), but no difference at V5 (−0.87 (−1.95; 0.22), *p* = 0.117).

According to CGI-S, iCBIT was associated with a greater reduction of disease severity compared to placebo at V3 (−0.26 (−0.42; −0.10), *p* = 0.001), V4 (−0.43 (−0.62; −0.25), *p* < 0.001), and V5 (−0.20 (−0.40; −0.01), *p* = 0.044).

For disease-specific quality of life (measured with the GTS-QoL), significant differences between iCBIT and placebo were observed at V4 (*p* = 0.002) and V5 (*p* = 0.013).

When assessing premonitory urges (PUTS-9), overall quality of life (GTS-QoL-VAS), and comorbidities (Y-BOCS, CAARS, BDI-II, BAI), we found ambiguous results with no consistent differences between iCBIT and placebo (for further details, please refer to Table 4).

No considerable differences were found at V3 and follow-up (V4, V5) between iCBIT, placebo, and f2f CBIT groups regarding the quality of the therapeutic alliance as assessed by WAI-SR (for further details, refer to Table S1).

### 3.5. Safety Analysis 

A total of 128 incidents were documented, with the lowest rate in the iCBIT group with 32.8% of patients with at least one incident compared to 34.3% in the placebo, and 58.3% in the f2f CBIT group with no significant difference between iCBIT and placebo (*p* = 0.857) but a significant difference between iCBIT and f2f CBIT (*p* = 0.028). None of the incidents were assessed as being treatment-related.

## 4. Discussion

In this multicenter, randomized, controlled, observer-blind trial, we investigated treatment effects of iCBIT (Minddistrict GmbH, 10117 Berlin, Germany), an internet-delivered, completely therapist-independent CBIT intervention, on tics in adult patients with CTD/TS compared to placebo and f2f CBIT. Our data suggest a clinically relevant tic reduction in adults with CTD/TS after iCBIT. Although the primary endpoint was narrowly missed, several secondary endpoints clearly indicate that iCBIT is superior compared to placebo in the treatment of tics. Remarkably, treatment effects of iCBIT further increased after end of treatment over a 6 month follow-up period.

Despite having missed statistical significance by a small margin, the primary analysis indicated a very strong trend towards superiority of iCBIT over placebo. Moreover, responder analyses showed a significant difference between iCBIT and placebo 1 week after end of treatment (V3), supporting the idea that iCBIT may be superior over placebo in the treatment of tics. In addition, we observed a potential tic improvement after iCBIT compared to placebo according to patients’ self-assessment (ATQ) and an improvement in quality of life (GTS-QoL).

Overall, our data are in line with recent studies showing a significant tic reduction in children and adolescents with CTD/TS after using internet-delivered CBIT/HRT interventions [26,27,28,29]. However, compared to these studies, we found a smaller tic reduction as assessed by YGTSS-TTS. This difference could be explained in several ways. First, our sample size was much larger (*n* = 161 versus *n* = 20 [26], *n* = 20 [27], *n* = 23 [28], and *n* = 41 [29]) and thus represents the largest study investigating CBIT/HRT in patients with CTD/TS. Second, we included an adult population, while in all other studies [26,27,28,29], children and adolescents were examined. Third, while iCBIT was designed as a completely therapist-independent CBIT intervention, in all recent studies [26,27,28,29], at least some support was provided by a therapist and/or parents/caregivers. For example, in the studies of Himle et al. (2012) and Ricketts et al. (2016), CBIT was delivered by a therapist via video instead of f2f [26,27]; in the study of Andrén et al. (2018), internet-delivered CBIT was accompanied by both therapists and parents [28]; and in the study of Rachamim et al. (2020), a therapist not only guided participants in the use of the online platform, but also repeatedly reminded and praised them to complete relevant modules and for making progress [29]. Furthermore, additional information and instructions specifically for parents/caregivers were integrated in the platform, enabling them to further support the children/adolescents in the best way possible [29]. In contrast, for iCBIT, only technical support, but no therapeutic support was provided at all. Due to blinding, patients were also not allowed to ask any questions related to iCBIT at clinic visits. In line with results obtained from psychotherapy research [30], it can be assumed that social reinforcement by a therapist further improves treatment effects also in the treatment of tics using CBIT, particularly since many patients perceive tic suppression and competing response training as exhausting [31]. Similar effects with decreased adherence and efficacy have also been shown in other mental disorders such as depression when using completely self-guided (internet-delivered) treatments [32,33,34,35]. Besides general support, reminder/request to complete homework assignments might play a crucial role in HRT/CBIT, since homework adherence has been found as a predictor for therapeutic response to CBIT [36]. Thus, we are convinced that our results did not reach significance, because in our sample size calculation, we neglected reduced treatment effects due to internet-delivered treatment (compared to f2f treatment). In other words, inclusion of a slightly higher number of patients would presumably have resulted in significant results.

Interestingly, only recently, Hollis et al. [37] reported the results of a randomized controlled study investigating a therapist-supported online remote exposure and response prevention training (ERP) in *n* = 224 children and adolescents with CTD/TS and examined effects on tics compared to psychoeducation after a 3 month treatment period using the YGTSS-TTS. Compared to our results at 3 months after end of treatment, they observed a nearly identical estimated mean difference in tic reduction between ERP and placebo (−2.29 [−3.86; 0.71] vs. −2.25 [−3.75; −0.75]). Despite several differences between the study of Hollis et al. [37] and our study (e.g., length of treatment period, participants’ age), the nearly identical tic reduction after iCBIT and ERP, respectively, further supports the hypothesis that both behavioral interventions, HRT/CBIT and ERP, are comparably effective [38].

In contrast to studies using f2f CBIT [6,7], in this study, treatment effects of iCBIT increased after end of treatment (at follow-up visits V4 and V5), resulting in a high tic reduction according to YGTSS-TTS compared to placebo 6 months after the end of treatment. It can be speculated that patients after having familiarized themselves with the iCBIT platform develop a greater self-responsibility while practicing the therapist-independent internet-delivered “self-treatment” iCBIT compared to classical f2f CBIT, resulting in continued training, even after the end of the actual treatment phase. In this study, patients in the iCBIT group could access the platform, not only until the individual end of treatment (V3), but until the end of the follow-up period (V5). Thus, it can be hypothesized that competing response training as the core element of HRT is the more effective option and can be broadened to more tics the longer it is practiced.

Although descriptively there was a larger tic reduction as assessed by YGTSS-TTS after f2f CBIT compared to iCBIT, results of this study indicate non-inferiority of iCBIT in comparison to f2f CBIT. At the same time, tic reduction was not significantly different in the f2f CBIT group compared to placebo, which might be best explained by the considerably smaller sample size of the f2f CBIT group (*n* = 24) compared to the placebo group (*n* = 70) with an associated large variation. However, when comparing treatment effects of f2f CBIT in this study to those in other controlled trials investigating efficacy of f2f HRT/CBIT in patients with CTD/TS [6,7,9], tic reduction observed in this study was smaller. We believe that the relatively small effect of f2f CBIT in this study was mainly related to patients’ expectations: the majority of patients who decided to participate in the study were interested in the online intervention and hoped to be randomized to the iCBIT group. Therefore, it can be assumed that most of those patients randomized to the f2f CBIT group were only poorly motivated, since expectations were not fulfilled. In addition, for some patients, f2f CBIT was associated with very long travel distances, because f2f CBIT was performed only in one center (MHH). Since we offered use of the iCBIT platform after end of treatment only to those patients in the f2f CBIT and placebo group who completed treatment and study visits, it can be speculated that some patients simply “sit out” f2f CBIT sessions in order to being allowed to access the iCBIT platform afterwards. Thus, it can be assumed that in studies primarily investigating effects of f2f CBIT/HRT (compared to placebo), patients are better motivated for f2f CBIT than in a study comparing efficacy of iCBIT to f2f CBIT.

In summary, on the basis of available data from both internet- and f2f-delivered CBIT, it can be speculated that a combination of both variants may result in largest tic reduction.

As expected, we found no treatment-related safety signals, neither for iCBIT nor for f2f CBIT.

The following limitations have to be addressed: (i) the dropout rate was relatively high, particularly in the iCBIT and the f2f CBIT groups. While dropouts in the f2f CBIT group may be best explained by poor motivation and unmet expectations, as outlined above, dropouts in the iCBIT group may be related to the fact that no guidelines and support by a therapist were provided, which may have been too burdensome for some patients. A meta-analysis examining self-guided psychological treatment for depressive symptoms indicate that adherence in completely self-guided treatments is considerably lower than in treatments with support [35]. In line with this assumption, studies investigating f2f CBIT showed lower dropout rates compared to this study [6,7]. Finally, some patients reported that the time required for iCBIT was greater than expected. We do not believe that the relatively high dropout rate had a relevant impact in favor towards iCBIT, since missing values were assumed to be missing-at-random (in the mixed model) or were replaced conservatively. In addition, sensitivity analyses ensured that the effect of iCBIT was not overestimated; (ii) the sample size of the f2f CBIT group was smaller than that of the iCBIT and the placebo groups, since f2f CBIT could only be offered at one single center in Hanover, which limited statistical analyses, since this study was not powered for the key secondary non-inferiority analysis; and (iii) since in general participants in studies investigating psychotherapeutic interventions are actively involved in the therapy [39], double-blinding was impossible. However, since several precautions were undertaken to keep the raters blind, we believe that our data are not biased by unintentional unblinding of the raters.

The following strengths of our study can be highlighted: (i) this is the largest study investigating the effects of CBIT/HRT on tics; (ii) compared to recent studies investigating effects of video-/internet-delivered CBIT/HRT on tics, this is not only the first study in adults, but also the first study investigating a completely therapist-independent approach; and (iii) this is the first study comparing directly iCBIT not only to placebo, but also to f2f CBIT.

## 5. Conclusions

Although the primary endpoint of the study was narrowly missed, our data clearly suggest that a completely therapist-independent internet-delivered CBIT is effective in the treatment of tics. However, it can be assumed that additional support from a therapist results in increased treatment effects. Since f2f CBIT often cannot be offered to patients, iCBIT may fill this gap to avoid pharmacotherapy with antipsychotics as first treatment option. Remarkably and in contrast to f2f CBIT, the treatment effect of iCBIT increased after “end of treatment”, suggesting that iCBIT results in greater self-responsibility once accepted by the patient. Finally, iCBIT is an extremely cost-effective treatment [40]. Thus, a combination of f2f and internet-delivered CBIT may result in greatest treatment effects.

## Figures and Tables

**Table 1:** Baseline characteristic: socio-demographic variables.

Clinical Data	iCBIT	Placebo (pl)	f2f CBIT (f2f)	Total			
	*n* = 67	*n* = 70	*n* = 24	*n* = 161	iCBIT vs. pl	iCBIT vs. f2f	pl vs. f2f
**Sociodemografic variable**					***p*-Value ^1^**		
**Age, mean (SD)**	34.64 (12.0%)	36.90 (13.2%)	34.92 (11.73%)	35.66 (12.47%)	0.297	0.923	0.516
**Male gender, *n* (%)**	45 (67.2%)	49 (70.0%)	18 (75.0%)	112 (69.6%)	0.721	0.475	0.640
**Education, *n* (%)**							
Baccalaureate	30 (44.8%)	25 (35.7%)	11 (45.8%)	66 (41.0%)	0.638	0.909	0.709
Secondary education	22 (32.8%)	25 (35.7%)	9 (37.5%)	56 (34.8%)			
Primary/main education	8 (11.9%)	10 (14.3%)	3 (12.5%)	21 (13.0%)			
Other	7 (6.7%)	10 (14.3%)	1 (4.2%)	18 (11.2%)			
**Employment, *n* (%)**							
Civil servant	2 (3.8%)	1 (1.9%)	0 (0.0%)	3 (2.4%)	0.583	0.629	0.818
Full-time	30 (57.7%)	28 (51.9%)	12 (60.0%)	70 (55.6%)			
Part-time	9 (17.3%)	7 (13.0%)	3 (15.0%)	19 (15.1%)			
Pensioner	0 (0.0%)	2 (3.7%)	1 (5.0%)	3 (2.4%)			
Disability pension	3 (5.8%)	3 (5.6%)	1 (5.0%)	7 (5.6%)			
Unemployed	6 (11.5%)	7 (13.0%)	3 (15.0%)	16 (12.7%)			
**Family status, *n* (%)**							
Married/civil union	20 (29.9%)	22 (31.4%)	6 (25.0%)	51 (31.7%)	0.813	0.891	0.807
Separated/divorced	6 (9.0%)	4 (5.7%)	3 (12.5%)	13 (8.1%)			
Single/unmarried	40 (59.7%)	43 (61.4%)	15 (62.5%)	98 (60.9%)			
Other	1 (1.5%)	1 (1.4%)	0 (0.0%)	2 (1.2%)			
**Living conditions, *n* (%)**							
Living alone	13 (19.4%)	18 (25.7%)	7 (29.2%)	38 (23.6%)	0.805	0.651	0.963
Living with relatives	40 (59.7%)	37 (52.9%)	10 (41.7%)	87 (54.0%)			
Living with parents	8 (11.9%)	10 (14.3%)	4 (16.7%)	22 (13.7%)			
Other	1 (1.5%)	1 (1.4%)	1 (4.2%)	3 (1.9%)			

iCBIT: Internet-delivered Comprehensive Behavioral Intervention for Tics; f2f CBIT: face-to-face Comprehensive Behavioral Intervention for Tics; ^1^ chi^2^-test was used for categorical variables and *t*-test for continuous variables.

**Table 2:** Baseline characteristic: clinical assessments.

	iCBIT	Placebo (pl)	f2f CBIT (f2f)	Total			
	*n* = 67	*n* = 70	*n* = 24	*n* = 161	iCBIT vs. pl	iCBIT vs. f2f	pl vs. f2f
Assessment	Mean (SD)				*p*-Value		
YGTSS-TTS	24.28 (7.40)	24.16 (8.36)	25.25 (9.44)	24.37 (8.10)	0.926	0.612	0.594
- MTS	15.12 (4.14)	14.53 (3.58)	15.83 (3.62)	14.97 (3.38)	0.372	0.457	0.128
- VTS	9.16 (5.27)	9.63 (6.36)	9.42 (6.49)	9.40 (5.92)	0.643	0.851	0.889
- impairment	25.97 (10.60)	24.99 (11.78)	23.33 (12.04)	25.15 (11.31)	0.609	0.316	0.557
- GSS	50.25 (16.15)	49.43 (16.59)	48.58 (17.15)	49.65 (16.40)	0.771	0.670	0.831
ATQ	57.70 (28.09)	48.97 (27.97)	54.86 (27.03)	53.60 (27.98)	0.974	0.876	0.895
MRVS	12.22 (3.88)	12.38 (3.61)	11.05 (4.25)	12.10 (3.83)	0.814	0.250	0.175
CGI-S	4.78 (0.78)	4.71 (0.76)	4.79 (0.78)	4.75 (0.77)	0.639	0.933	0.671
PUTS-9	21.05 (5.59)	20.46 (6.44)	19.71 (5.58)	20.59 (5.96)	0.571	0.318	0.613
GTS-QoL	21.98 (15.90)	23.39 (16.53)	27.90 (14.44)	23.47 (16.00)	0.616	0.120	0.248
GTS-QoL-VAS	63.91 (18.84)	62.16 (21.19)	54.96 (24.07)	61.81 (20.79)	0.612	0.067	0.170
BDI-II	10.16 (9.88)	11.36 (10.05)	12.17 (9.67)	10.98 (9.89)	0.485	0.394	0.734
BAI	10.63 (10.23)	9.48 (8.27)	12.70 (10.08)	10.43 (9.40)	0.472	0.403	0.130
CAARS: ADHD Index	9.50 (5.42)	11.52 (6.15)	12.59 (5.57)	10.84 (5.87)	0.129	0.046 *	0.517

iCBIT: internet-delivered Comprehensive Behavioral Intervention for Tics; f2f CBIT: face-to-face Comprehensive Behavioral Intervention for Tics; YGTSS-MTS: Yale Global Tic Severity Scale-Motor Tic Score; YGTSS-VTS: Yale Global Tic Severity Scale-Vocal Tic Score; YGTSS-TTS: Yale Global Tic Severity Scale-Total Tic Score; YGTSS-impairment: Yale Global Tic Severity Scale-Impairment Score; YGTSS-GSS: Yale Global Tic Severity Score-Global Severity Score; ATQ: Adult Tic Questionnaire; MRVS: Modified Rush Video-Based Tic Rating Scale; CGI-S: Clinical Global Impression-Severity; PUTS-9: Premonitory Urge for Tics Scale; GTS-QoL: Gilles de la Tourette Syndrome Quality of Life Scale; GTS-QoL-VAS: Quality of Life-Visual Analogue Scale; BDI-II: Beck Depression Inventory-II; BAI: Beck Anxiety Inventory; CAARS: Conners’ Adult ADHD Rating Scales; ADHD: Attention Deficit and Hyperactivity Disorder; Y-BOCS: Yale Brown Obsessive Compulsive Scale; * *p* < 0.05; bold: trends, *p* < 0.10.

**Table 3:** Comparisons of YGTSS-TTS and YGTTS subscales between iCBT, placebo, and f2f CBIT at 1 week after end of treatment (V3, primary endpoint) and follow-up visits (V4, V5).

Group 1 vs. 2	Variable (Change-to-Baseline)	Visit	Group 1: LS Mean (95% CI)	Group 2: LS Mean (95% CI)	LS Mean Diff Arm 1–2 (95% CI)	*p*-Value
iCBIT vs. placebo	YGTSS-TTS	V3	−2.54 (−3.53; −1.55)	−1.26 (−2.16; −0.35)	−1.28 (−2.58; 0.01)	**0.053**
		V4	−3.69 (−4.84; −2.54)	−1.44 (−2.48; −0.40)	−2.25 (−3.75; −0.75)	0.003 *
		V5	−4.27 (−5.47; −3.07)	−1.56 (−2.63; −0.48)	−2.71 (−4.27; −1.16)	<0.001 *
	YGTSS-MTS	V3	−1.31 (−1.84; −0.77)	−0.42 (−0.91; 0.07)	−0.89 (−1.59; −0.19)	0.013 *
		V4	−2.29 (−2.91; −1.68)	−0.41 (−0.97; 0.14)	−1.88 (−2.68; −1.08)	<0.001 *
		V5	−2.62 (−3.28; −1.96)	−0.91 (−1.50; −0.32)	−1.71 (−2.56; −0.86)	<0.001 *
	YGTSS-VTS	V3	−1.26 (−1.95; −0.56)	−0.86 (−1.50; −0.23)	−0.40 (−1.31; 0.52)	0.395
		V4	−1.51 (−2.31; −0.71)	−1.05 (−1.78; −0.33)	−0.46 (−1.50; 0.59)	0.390
		V5	−1.69 (−2.52; −0.85)	−0.69 (−1.42; 0.05)	−1.00 (−2.08; 0.07)	**0.068**
	YGTSS- Impairment	V3	−5.20 (−7.39; −3.01)	−1.77 (−3.78; 0.24)	−3.43 (−6.29; −0.56)	0.019 *
		V4	−6.32 (−8.84; −3.79)	−3.52 (−5.83; −1.22)	−2.80 (−6.10; 0.51)	**0.097**
		V5	−6.38 (−9.16; −3.60)	−1.25 (−3.70; 1.21)	−5.13 (−8.72; −1.54)	0.005 *
iCBIT vs. f2f CBIT	YGTSS-TTS	V3	−2.71 (−3.74; −1.68)	−3.69 (−5.54; −1.83)	0.98 (−1.01; 2.96)	0.333
		V4	−3.93 (−5.12; −2.73)	−5.74 (−7.96; −3.53)	1.82 (−0.54; 4.18)	0.130
		V5	−4.48 (−5.76; −3.20)	−4.73 (−7.04; −2.42)	0.25 (−2.23; 2.73)	0.841
	YGTSS-MTS	V3	−1.45 (−2.04; −0.86)	−2.26 (−3.34; −1.17)	0.81 (−0.34; 1.95)	0.166
		V4	−2.46 (−3.13; −1.79)	−2.82 (−4.07; −1.56)	0.35 (−0.97; 1.68)	0.601
		V5	−2.77 (−3.51; −2.04)	−2.62 (−3.96; −1.28)	−0.15 (−1.57; 1.28)	0.838
	YGTSS-VTS	V3	−1.28 (−1.99; −0.57)	−1.53 (−2.79; −0.27)	0.25 (−1.11; 1.61)	0.717
		V4	−1.56 (−2.38; −0.75)	−3.06 (−4.56; −1.56)	1.50 (−0.12; 3.12)	**0.070**
		V5	−1.74 (−2.61; −0.88)	−2.24 (−3.76; −0.72)	0.50 (−1.16; 2.16)	0.556
	YGTSS- Impairment	V3	−5.55 (−7.84; −3.25)	−5.97 (−10.16; −1.78)	0.43 (−4.04; 4.89)	0.851
		V4	−6.73 (−9.34; −4.12)	−5.61 (−10.51; −0.70)	−1.13 (−6.35; 4.10)	0.672
		V5	−6.76 (−9.59; −3.94)	−5.82 (−10.92; −0.72)	−0.95 (−6.44; 4.54)	0.734
placebo vs. f2f CBIT	YGTSS-TTS	V3	−1.09 (−2.00; −0.18)	−2.35 (−4.10; −0.59)	1.26 (−0.59; 3.10)	0.180
		V4	−1.23 (−2.25; −0.21)	−3.71 (−5.74; −1.68)	2.48 (0.36; 4.60)	0.022 *
		V5	−1.38 (−2.44; −0.32)	−2.71 (−4.82; −0.61)	1.33 (−0.85; 3.52)	0.230
	YGTSS-MTS	V3	−0.34 (−0.84; 0.15)	−1.46 (−2.41; −0.51)	1.12 (0.13; 2.11)	0.027 *
		V4	−0.33 (−0.89; 0.24)	−1.83 (−2.95; −0.70)	1.50 (0.33; 2.67)	0.012 *
		V5	−0.85 (−1.47; −0.24)	−1.72 (−2.92; −0.51)	0.86 (−0.39; 2.12)	0.177
	YGTSS-VTS	V3	−0.80 (−1.41; −0.18)	−0.95 (−2.13; 0.23)	0.15 (−1.09; 1.40)	0.808
		V4	−0.95 (−1.62; −0.27)	−1.95 (−3.28; −0.61)	1.00 (−0.40; 2.40)	0.161
		V5	−0.58 (−1.27; 0.11)	−1.06 (−2.41; 0.29)	0.48 (−0.94; 1.90)	0.506
	YGTSS- Impairment	V3	−1.20 (−3.08; 0.67)	−4.60 (−8.17; −1.02)	3.39 (−0.38; 7.17)	**0.077**
		V4	−2.90 (−5.13; −0.66)	−3.10 (−7.53; 1.33)	0.20 (−4.46; 4.87)	0.931
		V5	−0.57 (−2.97; 1.82)	−3.11 (−7.77; 1.55)	2.54 (−2.42; 7.49)	0.314

iCBIT: Internet-delivered Comprehensive Behavioral Intervention for Tics; f2f CBIT: face-to-face Comprehensive Behavioral Intervention for Tics; YGTSS-MTS: Yale Global Tic Severity Scale-Motor Tic Score; YGTSS-VTS: Yale Global Tic Severity Scale-Vocal Tic Score; YGTSS-TTS: Yale Global Tic Severity Scale-Total Tic Score; YGTSS-impairment: Yale Global Tic Severity Scale-Impairment Score; LS: least square; Diff: difference; CI: confidence interval; V: visit; * *p* < 0.05; bold: trends, *p* < 0.10; negative values favor group 1.

**Table 4:** Comparison of further assessments for tics, comorbidities, and quality of life between iCBIT and placebo at 1 week after end of treatment (V3, primary endpoint) and follow-up visits (V4, V5).

Assessment (CFB)	Visit	iCBIT: LS Mean (95% CI)	Placebo: LS Mean (95% CI)	LS Mean Diff iCBIT-pl (95% CI)	*p*-Value
ATQ	V3	−7.50 (−11.62; −3.38)	−1.32 (−5.04; 2.40)	−6.18 (−11.57; −0.80)	**0.025**
	V4	−10.42 (−14.65; −6.19)	−3.25 (−7.23; 0.73)	−7.17 (−12.78; −1.55)	**0.013**
	V5	−7.80 (−12.35; −3.24)	−1.54 (−5.76; 2.69)	−6.26 (−12.27; −0.25)	**0.041**
CGI-S	V3	−0.41 (−0.53; −0.29)	−0.15 (−0.26; −0.03)	−0.26 (−0.42; −0.10)	0.001 *
	V4	−0.60 (−0.75; −0.46)	−0.17 (−0.30; −0.04)	−0.43 (−0.62; −0.25)	<0.001 *
	V5	−0.48 (−0.63; −0.32)	−0.27 (−0.41; −0.14)	−0.20 (−0.40; −0.01)	0.044 *
MRVS	V3	−0.94 (−1.66; −0.23)	−0.06 (−0.75; 0.63)	−0.88 (−1.84; 0.08)	0.072
	V4	−1.83 (−2.58; −1.08)	−0.53 (−1.14; 0.28)	−1.40 (−2.40; −0.40)	0.006 *
	V5	−1.11 (−1.91; −0.30)	−0.24 (−1.01; 0.53)	−0.87 (−1.95; 0.22)	0.117
PUTS-9	V3	0.33 (−0.55; 1.20)	−1.13 (−1.92; −0.34)	1.46 (0.32; 2.59)	0.012 *
	V4	0.32 (−0.67; 1.31)	−0.45 (−1.35; 0.45)	0.77 (−0.51; 2.06)	0.238
	V5	−0.07 (−1.18; 1.04)	−0.58 (−1.56; 0.40)	0.51 (−0.92; 1.93)	0.484
GTS-QoL	V3	−3.58 (−5.87; −1.28)	−1.90 (−4.01; 0.20)	−1.67 (−4.67; 1.32)	0.272
	V4	−6.95 (−9.57; −4.32)	−1.66 (−4.04; 0.72)	−5.28 (−8.69; −1.88)	0.002 *
	V5	−5.86 (−8.66; −3.05)	−1.26 (−3.76; 1.24)	−4.60 (−8.21; −0.99)	0.013 *
GTS-QoL-VAS	V3	−3.49 (−7.05; 0.06)	3.83 (0.57; 7.09)	−7.32 (−12.02; −2.62)	0.002 *
	V4	3.80 (−0.08; 7.68)	2.11 (−1.43; 5.65)	1.69 (−3.44; 6.82)	0.517
	V5	−1.38 (−5.76; 3.01)	4.31 (0.45; 8.17)	−5.68 (−11.37; 0.00)	**0.050**
Y-BOCS	V3	−1.57 (−2.48; −0.66)	0.29 (−0.57; 1.14)	−1.86 (−3.05; −0.67)	0.002 *
	V4	−0.89 (−1.98; 0.20)	−0.66 (−1.66; 0.33)	−0.23 (−1.63; 1.18)	0.752
	V5	−1.37 (−2.51; −0.23)	−0.50 (−1.51; 0.52)	−0.87 (−2.33; 0.58)	0.239
CAARS: ADHD Index	V3	−0.98 (−1.83; −0.13)	−0.32 (−1.06; 0.42)	−0.66 (−1.73; 0.41)	0.227
	V4	−1.85 (−2.81; −0.90)	−0.43 (−1.25; 0.38)	−1.42 (−2.63; −0.21)	0.021 *
	V5	−0.97 (−2.03; 0.10)	0.05 (−0.82; 0.93)	−1.02 (−2.34; 0.30)	0.129
BDI-II	V3	−1.98 (−3.51; −0.45)	−0.95 (−2.36; 0.46)	−1.03 (−3.04; 0.98)	0.312
	V4	−3.36 (−4.97; −1.75)	−1.13 (−2.60; 0.34)	−2.23 (−4.33; −0.12)	0.038 *
	V5	−1.85 (−3.58; −0.13)	−0.77 (−2.35; 0.81)	−1.09 (−3.34; 1.17)	0.345
BAI	V3	−1.10 (−2.45; 0.24)	1.06 (−0.18; 2.30)	−2.17 (−3.94; −0.39)	0.017 *
	V4	−1.90 (−3.38; −0.42)	0.97 (−0.38; 2.32)	−2.87 (−4.81; −0.93)	0.004 *
	V5	−1.58 (−3.24; 0.09)	−0.16 (−1.66; 1.34)	−1.42 (−3.58; 0.75)	0.198

ATQ: Adult Tic Questionnaire; MRVS: Modified Rush Video-Based Tic Rating Scale; CGI-S: Clinical Global Impression-Severity; PUTS-9: Premonitory Urge for Tics Scale; GTS-QoL: Gilles de la Tourette Syndrome Quality of Life Scale; GTS-QoL-VAS: Quality of Life-Visual Analogue Scale; BDI-II: Beck Depression Inventory-II; BAI: Beck Anxiety Inventory; CAARS: Conners’ Adult ADHD Rating Scales; ADHD: Attention Deficit and Hyperactivity Disorder; Y-BOCS: Yale Brown Obsessive Compulsive Scale; CFB: Change from Baseline; * *p* < 0.05; bold: trends, *p* < 0.10.

## Data Availability

The data presented in this study are available on request from the corresponding author (K.M.-V.).

## Supplementary Materials

The following are available online at https://www.mdpi.com/article/10.3390/jcm11010250/s1, Figure S1: Course of YGTSS-MTS across all visits in all study groups. Figure S2: Course of YGTSS-VTS across all visits in all study groups. Figure S3: Course of YGTSS-impairment across all visits in all study groups. Table S1: Estimates for therapeutic alliance (according to WAI-SR) by treatment and visit—linear regression model (stratified for center and anti-tic medication).
