# Supplementary material for: ONLINE-TICS: Internet-Delivered Behavioral Treatment for Patients with Chronic Tic Disorders
Source: J Clin Med. 2022 Jan 4;11(1):250. doi: 10.3390/jcm11010250 (PMC8745756; doi:10.3390/jcm11010250)
Supplement: Supplementary file 1 [file jcm-11-00250-s001.zip › jcm-1513234-supplementary.pdf]

Journal of
Clinical Medicine

MDPI

Supplementary Material

Table S1. Estimates for therapeutic alliance (according to WAI-SR) by treatment and visit – linear regression model (stratified for center and tic medication)

Intervention group	Reference group	Visit	Cases included	Estimate (95%-CI)	*p*-value
iCBIT	placebo	V2	116/137	3.61 (0.13; 7.10)	**0.042**
		V3	118/137	2.17 (−1.80; 6.13)	0.281
		V4	118/137	3.09 (−0.66; 6.84)	0.106
		V5	118/137	3.36 (−0.43; 7.14)	0.082
iCBIT	f2f CBIT	V2	80/91	−0.16 (−6.34; 6.02)	0.959
		V3	80/91	−2.45 (−8.99; 4.09)	0.458
		V4	80/91	−2.36 (−8.92; 4.21)	0.476
		V5	80/91	−2.65 (−9.12; 3.81)	0.416
f2f CBIT	placebo	V2	82/94	−0.13 (−5.97; 5.71)	0.965
		V3	84/94	−1.93 (−8.35; 4.49)	0.551
		V4	84/94	−3.55 (−9.70; 2.60)	0.254
		V5	84/94	−3.39 (−9.67; 2.89)	0.285

iCBIT: internet-delivered Comprehensive Behavioral Intervention for Tics; f2f CBIT: face-to-face Comprehensive Behavioral Intervention for Tics; CI: Confidence Interval.

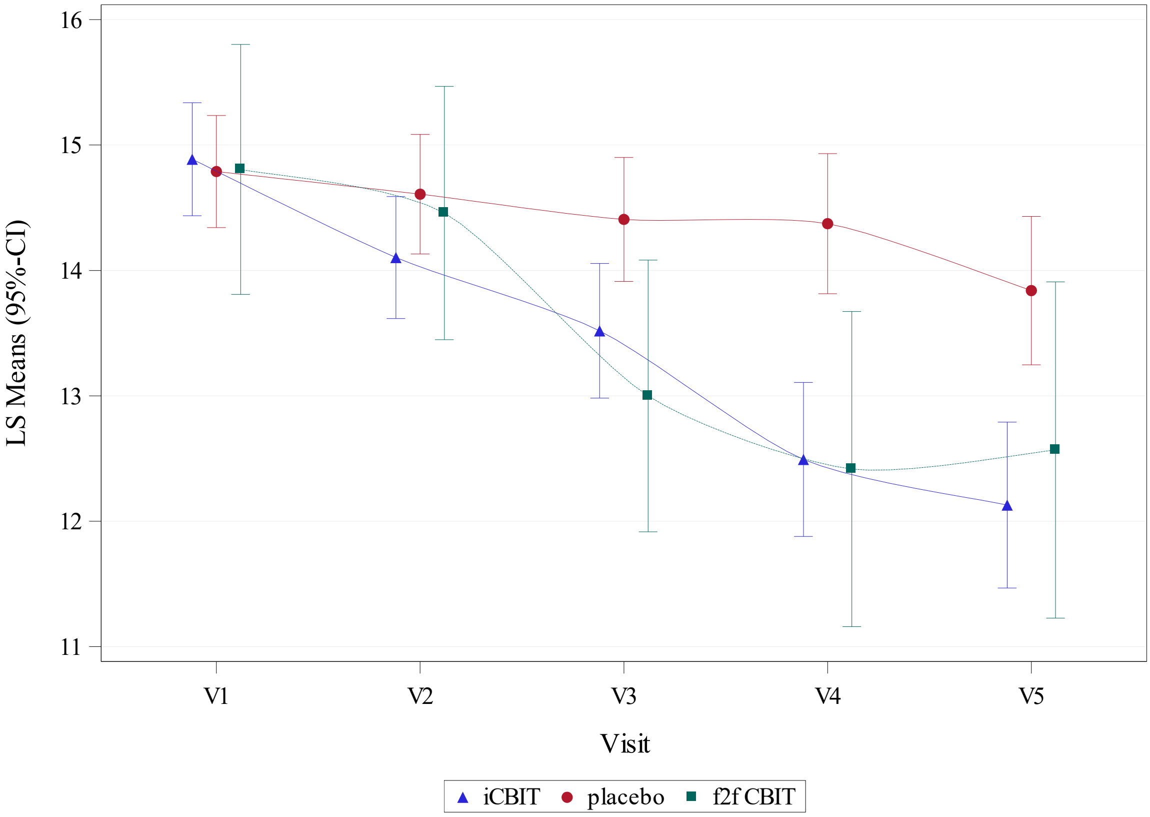

Figure S1. Course of YGTSS-MTS across all visits in all study arms. iCBIT: internet-delivered Comprehensive Behavioral Intervention for Tics; f2f CBIT: face-to-face Comprehensive Behavioral Inter-vention for Tics.

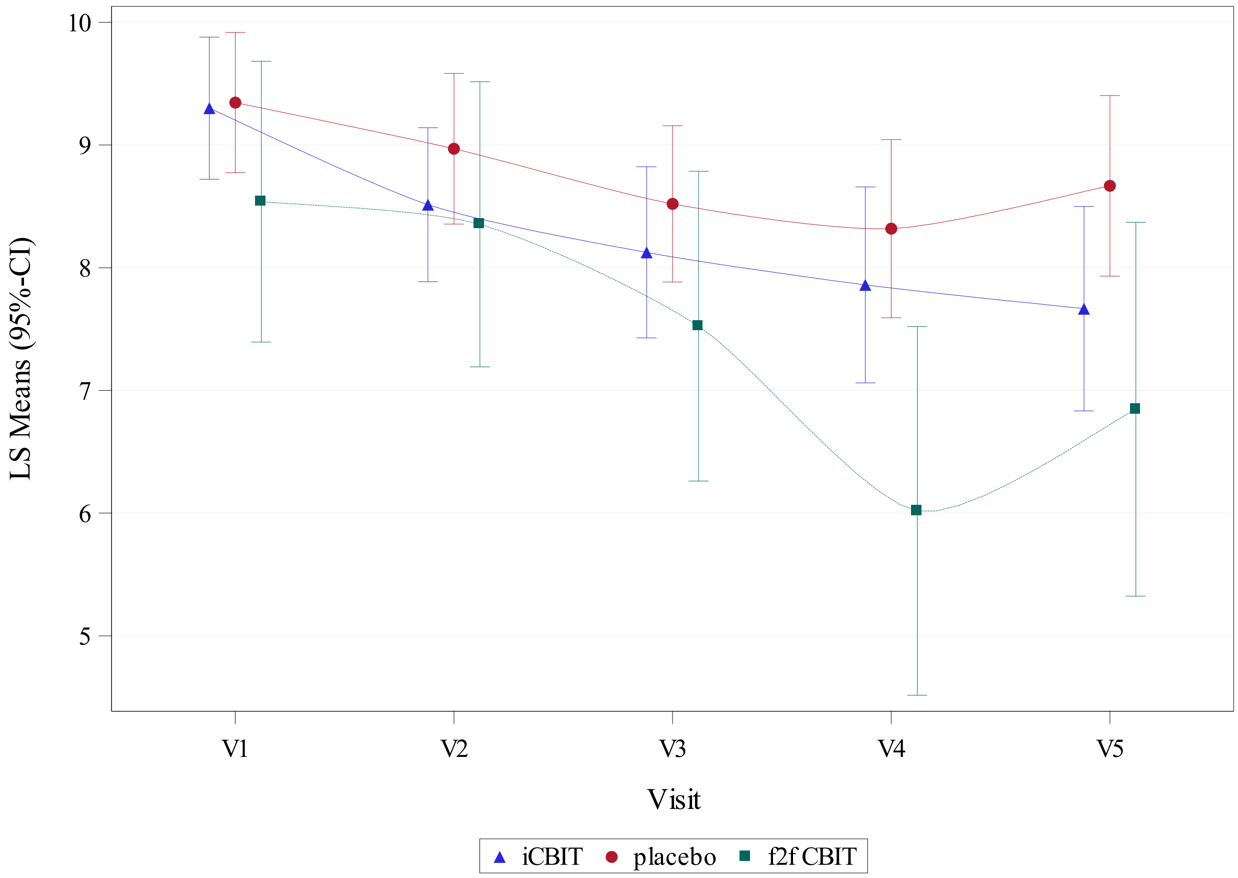

Figure S2. Course of YGTSS-VTS across all visits in all study arms.

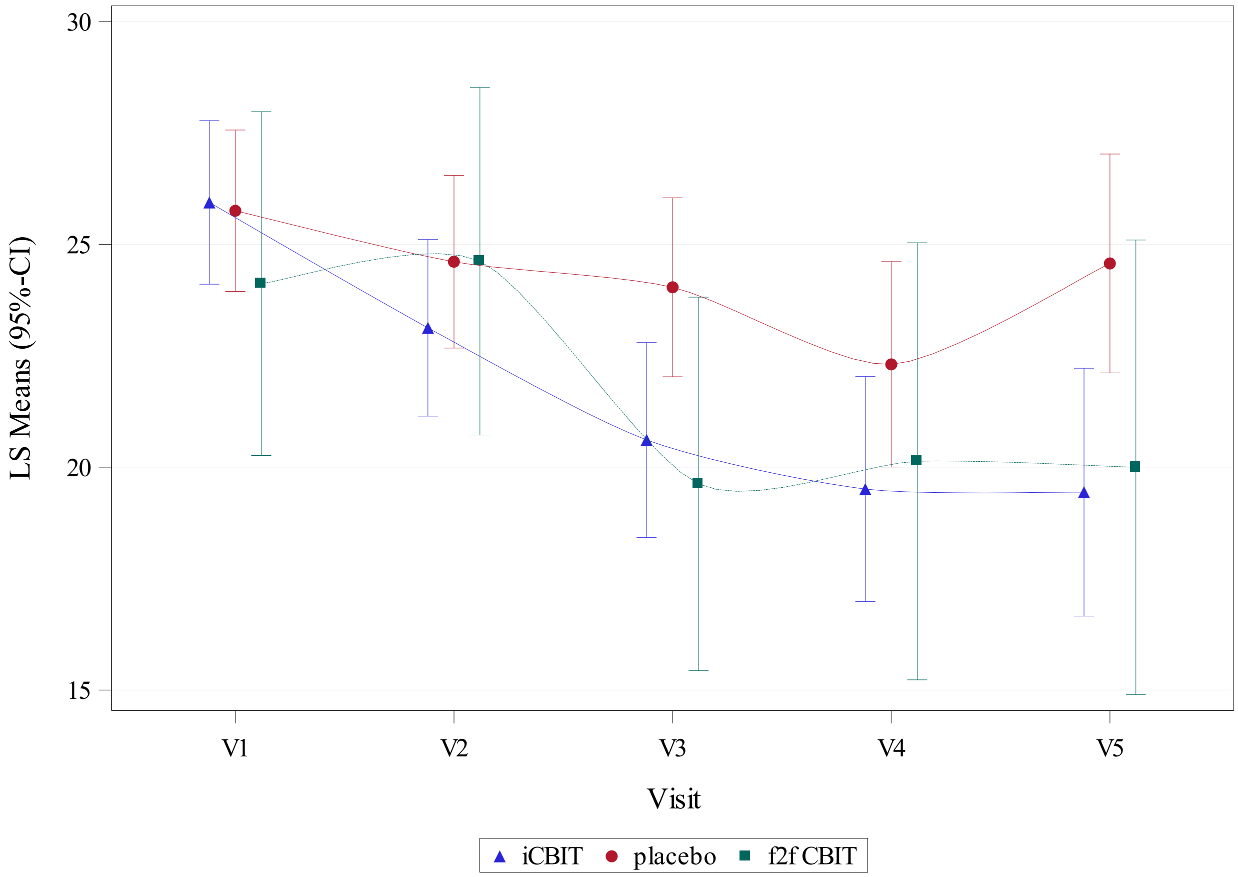

Figure S3. Course of YGTSS-impairment across all visits in all study arms.
